# Clinical Utility of a Novel Molecular Assay in Various Combination Strategies with Existing Methods for Diagnosis of HIV-Related Tuberculosis in Uganda

**DOI:** 10.1371/journal.pone.0107595

**Published:** 2014-09-15

**Authors:** Willy Ssengooba, Lydia Nakiyingi, Derek T. Armstrong, Frank G. Cobelens, David Alland, Yukari C. Manabe, Susan E. Dorman, Jerrold J. Ellner, Moses L. Joloba

**Affiliations:** 1 Department of Medical Microbiology, College of Health Sciences, Makerere University, Kampala, Uganda; 2 Infectious Diseases Institute, College of Health Sciences, Makerere University, Kampala, Uganda; 3 Johns Hopkins University School of Medicine, Baltimore, Maryland, United States of America; 4 Department of Global Health and Amsterdam Institute of Global Health and Development, Academic Medical Center, University of Amsterdam, Amsterdam, Netherlands; 5 KNCV Tuberculosis Foundation, The Hague, Netherlands; 6 Boston Medical Center, Boston University School of Medicine, Boston, Massachusetts, United States of America; National Institute of Infectious Diseases, Japan

## Abstract

**Background:**

Low income, high-tuberculosis burden, countries are considering selective deployment of Xpert MTB/RIF assay (Xpert) due to high cost per test. We compared the diagnostic gain of the Xpert add-on strategy with Xpert replacement strategy for pulmonary tuberculosis diagnosis among HIV-infected adults to inform its implementation.

**Methods:**

The first diagnostic sputum sample of 424 HIV-infected adults (67% with CD4 counts ≤200/mm^3^) suspected for tuberculosis was tested by direct Ziehl-Neelsen (DZN) and direct fluorescent microscopy (DFM); concentrated fluorescent microscopy (CFM); Lowenstein-Jensen (LJ) and Mycobacterial Growth Indicator Tube (MGIT) culture; and Xpert. Overall diagnostic yield and sensitivity were calculated using MGIT as reference comparator. The sensitivity of Xpert in an add-on strategy was calculated as the number of smear negative but Xpert positive participants among MGIT positive participants.

**Results:**

A total of 123 (29.0%) participants were MGIT culture positive for *Mycobacterium tuberculosis*. The sensitivity (95% confidence interval) was 31.7% (23.6–40.7%) for DZN, 35.0% (26.5–44.0%) for DFM, 43.9% (34.9–53.1%) for CFM, 76.4% (67.9–83.6) for Xpert and 81.3% (73.2–87.7%) for LJ culture. Add-on strategy Xpert showed an incremental sensitivity of 44.7% (35.7–53.9%) when added to DZN, 42.3% (33.4–51.5%) to DFM and 35.0% (26.5–44.0%) to CFM. This translated to an overall sensitivity of 76.4%, 77.3% and 79.0% for add-on strategies based on DZN, DFM and CFM, respectively, compared to 76.4% for Xpert done independently. From replacement to add-on strategy, the number of Xpert cartridges needed was reduced by approximately 10%.

**Conclusions:**

Among HIV-infected TB suspects, doing smear microscopy prior to Xpert assay in add-on fashion only identifies a few additional TB cases.

## Background

Tuberculosis (TB) remains the most important opportunistic infection causing death among HIV-infected individuals in Sub-Saharan Africa [1]. Microscopic examination of Ziehl-Neelsen (ZN)-stained sputum smears, the most commonly available diagnostic in resource-limited settings, has low sensitivity for TB detection, especially among HIV-infected individuals. Therefore, at least two sputum specimens need to be tested. However, in practice, the second smear is difficult to obtain for logistical reasons [2]. Fluorescence microscopy (FM) is 7–10% more sensitive for TB detection than conventional light microscopy [3]–[5], but the increase in sensitivity among HIV-infected individuals is often lower than in HIV-uninfected individuals [6]–[9].

Sputum culture, generally considered the gold standard for TB diagnosis, takes several weeks to yield results and may not be available before individuals are lost to follow-up or even dead [10]. The use of culture for diagnosing TB was recommended by the World Health Organization (WHO) among HIV-infected individuals [11]. However, it has not been widely scaled-up due to infrastructure and other resource requirements, shortages of qualified laboratory personnel, limited access to training for specific tests and logistical limitations, as well as longer time to detection [12].

The WHO recently endorsed use of a molecular-based diagnostic, the Xpert MTB/RIF assay (Xpert; Cepheid, Sunnyvale, CA, USA) as a frontline diagnostic for TB among HIV-infected individuals [13]. An all-in-one-cartridge real-time PCR, it offers a total run time of 2 hours, higher sensitivity, and simultaneous susceptibility results for rifampicin in one test run. However, this test is still under-utilized in high TB burden countries due to the investment cost of the equipment, maintenance costs and the individual cartridge cost [13], [14]. Furthermore, its sensitivity for diagnosing TB among smear-negative individuals ranges from 56–88% [15]–[18]; in some cases, TB culture is still required for diagnosis.

Although WHO now recommends that Xpert be used as the initial test in adults and children presumed to have HIV-associated TB [19], several low-income countries are adopting an add-on strategy in which smear microscopy is used first in individuals clinically suspected to have TB, and if the smear is negative and the individual is HIV-positive then the sputum is subsequently tested by Xpert [14], [20], [21]. This is partly based on considerations of affordability. Indeed, an economic analysis suggested that for a low-income country such as Uganda an add-on strategy had lower diagnostic and treatment costs than a replacement strategy in which Xpert was used as the first-line test [14]. However, this analysis did not take into account possible differences between HIV-positive and HIV-negative individuals, nor possible dropouts from the diagnostic process, which may occur if individuals first have to wait for smear results or come back. Furthermore, previous studies did not address the effects of testing only one sputum sample, or of using more sensitive smear microscopy methods [14], [21]. In Uganda, there were 26 health facilities with functional Xpert services by the end of June 2012 [22]. In financial year 2012/2013 the number of Xpert machines in Uganda increased to 45, of which 42 (93%) were fully functional and three were awaiting installation. This was against a National Tuberculosis Control Program target of 200 Xpert machines by the year 2015. At this Xpert roll-out rate, there will be about 38 Xpert machines in the coming two years leading to 83 operational Xpert centers by 2015. The national guidance is to utilize Xpert among smear negative HIV-infected individuals and for screening for rifampicin resistance among retreatment TB patients, due to reasons of affordability and challenges of optimal procurement of cartridges, in contrast to WHO recommendation [19]. There thus remains a need to assess the most effective diagnostic strategy that would be relevant for routine practice using systematically collected evidence for effectiveness before implementation [20], [23].

In this study, we compared the diagnostic gain of various Xpert add-on strategies with that of an Xpert replacement strategy for diagnosis of pulmonary TB among HIV-infected adults to inform its implementation in Uganda.

## Methods

### Study participants

This was a secondary data analysis from a prospective TB diagnostics study among HIV-infected participants suspected of TB disease [24]. It consisted of both inpatients and outpatients of Mulago National referral Hospital and the Infectious Diseases Institute (IDI) of Makerere University in Kampala, Uganda. The participants were HIV-infected TB suspects with at least one sign or symptom of TB. The enrollment period was January 2011 through November 2011. For the accuracy study, two sputum samples were collected for direct ZN, direct FM, concentrated FM and culture on both solid and liquid media. Blood samples for CD4 cell count and for culture were collected at study enrollment. For the present analysis, we considered a single sputum sample only, namely the sample submitted by the participant on the first diagnostic encounter.

### Laboratory procedures

Mycobacteriological laboratory procedures were done at the Mycobacteriology (BSL-3) laboratory which is under the Medical Microbiology department of Makerere University. All examinations were according to standard procedures for culture, direct and concentrated smears for microscopy [25]. Briefly smears were made from unprocessed sputum samples, stained using standard reagents and examined by light for direct ZN microscopy (DZN) or direct auramine O-stained FM (DFM) at ×100 and ×40 objectives respectively (Olympus CX31 with LED attachment, Olympus Corporation, Tokyo, Japan). Smear results were reported as scanty, 1+, 2+ and 3+ using the WHO grading system [26]. Positive results were issued within 24 hours to the requesting physicians to initiate TB treatment. The remaining sputum samples were processed by digestion and decontamination with NALC/NAOH 2% for 15 min, followed by dilution with phosphate buffer (PH 6.8) up to 45 ml. The homogenized sample was then centrifuged at 3000*g* for 15 min. The sediment was re-suspended with phosphate buffer (pH 6.8) to a volume of 2 ml, of which 0.5 ml was inoculated in MGIT and two LJ tubes per sample for mycobacterial culture. At this point, a smear from the suspension was made for concentrated FM (CFM). Residual aliquots were stored frozen at −80°C. Cultures on LJ were incubated at 37°c for up to 8 weeks and the MGIT in a machine for up to 6 weeks.

All cultures with growth were sub-cultured on blood agar to exclude contamination and a smear was examined by ZN smear microscopy. Specimen positive for acid-fact bacilli (AFB) underwent Capillia Neo TB (TAUN, Numazu, Japan) testing. Capillia positive specimens were classified as *Mycobacterium tuberculosis complex* (MTB) and those negative as Non-tuberculous Mycobacteria (NTM). Those that were AFB-negative and had growth on blood agar were classified as contaminated and those without growth throughout were classified as negative.

The Xpert assay was performed on the leftover sputum pellets from culture. Samples were thawed from −80°C to room temperature. Procedures for Xpert were done using a 1∶3 (sample: sample reagent) dilution. This was vigorously mixed and incubated at room temperature for 15 minutes and one mL of the mixture was transferred to the Xpert cartridge. The cartridge was then inserted into the Xpert machine; processing and result interpretation is automated, using software version 4.0.

### Statistical analysis

Data were double entered in an electronic database (MS-Access, Microsoft Corp, Seattle WA, USA); discrepancies were solved by checking the entries against the raw data. Data were exported to Stata v11 (Stata Corp, College Station TX, USA) for analysis. We used the exact binomial method for calculating 95% confidence intervals and the 2-sided Fisher's exact test for comparing proportions.

**Figure 1:**
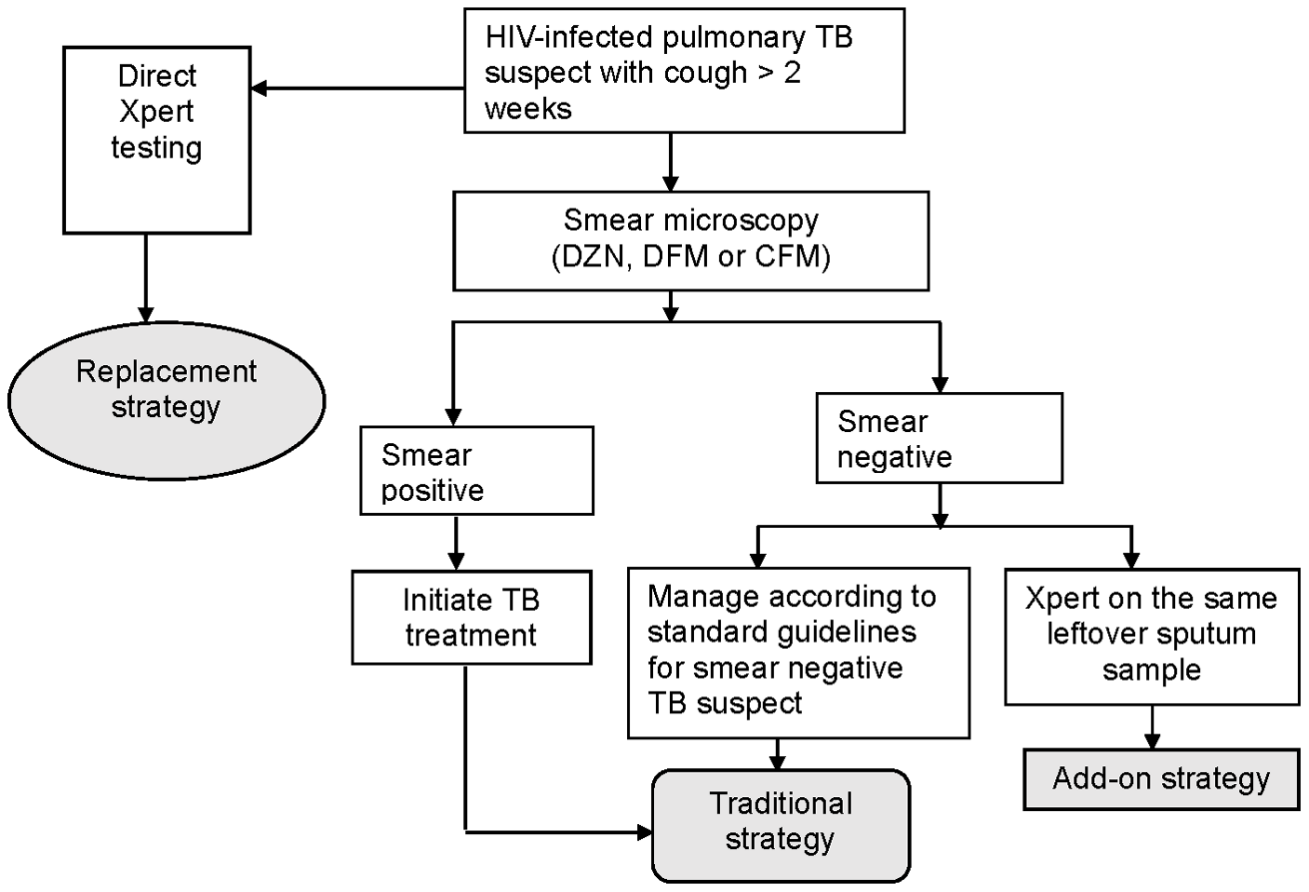
Hypothetical Xpert implementation in either a replacement or an add-on strategy. DZN  =  Direct Ziehl Neelsen, DFM  =  Direct Fluorescent Microscopy, CFM  =  Concentrated Fluorescent Microscopy, Xpert  =  Xpert MTB/RIF test.

Diagnostic yield was defined as the observed number of TB cases detected by each test employed. Sensitivity per test was calculated as the proportion positive using MGIT culture as the reference comparator. The incremental sensitivity for an add-on strategy of Xpert to smear microscopy was calculated as the number of participants positive by Xpert but negative on the smear microscopy method divided by the total number of TB cases detected by MGIT culture (Figure 1).

### Ethics statement

The study protocol was approved by Makerere University School of Public Health Institutional Review Board (MUSPHIRB) and the Uganda National Council of Science and Technology (UNCST). All participants gave written informed consent.

## Results

### Characteristics of participants

A total of 523 HIV-infected participants were screened for the study and 498 had the first sputum sample on the first diagnostic encounter. A total of 74 participants were excluded from this analysis: 19 (3.8%) due to LJ contamination, 46 (9.2%) due to MGIT contamination, and 9 (1.8%) due to contamination on both LJ and MGIT culture. There were no missing or invalid Xpert results, leaving 424 participants for the analysis. Table 1 shows the characteristics of participants included in this analysis.

**Table 1:** Characteristics and CD4 cell count categories of participants enrolled (N = 424).

Parameter	Number (%)
Female	269 (63.4)
Median age(IQR)	32 (32–34)
MGIT culture positive TB cases	123 (29.0)
**CD4 cell count among MGIT confirmed TB cases (N = 419)**	
**Total (N)**	**121 (28.9)**
Median CD4/mm^3^ (IQR)	67 (43–92)
CD4<50 (n = 164)	52 (31.7)
CD4 51–200 (n = 117)	45 (38.4)
CD4>200 (138)	24 (17.4)
**Grew NTM on MGIT culture**	**19 (4.5)**
CD4<50 (n = 164)	12 (7.3)
CD4 51–200 (n = 117)	3 (2.6)
CD4>200 (138)	4 (2.9)

**Key:** IQR  =  Inter Quartile Range, MGIT  =  Mycobacterial Growth Indicator Tube, NTM  =  Non Tuberculous Mycobacteria.

### Comparative performance of the methods used for diagnosis of HIV-related tuberculosis

Overall 123 (29.0%) of the participants were microbiologically confirmed TB cases by MGIT culture. Xpert was negative for two specimens with DZN positive smears (one graded scanty and one graded 1+) of which one was culture negative, for three specimens with DFM positive smears (all graded scanty) of which two were culture positive *for M. tuberculosis*, and for four specimens with CFM positive smears (one graded scanty, two 1+ and one 2+) of which three were *M. tuberculosis* culture positive. The total number of culture positive TB cases who were smear positive but Xpert negative were four of which DZN detected one, DFM detected two and CFM detected all those detected by DZN and DFM with additional two TB cases (Table S1).

Of the Xpert-positive specimens, four were resistant to rifampicin, also confirmed by phenotypic drug susceptibility testing using MGIT. These related to two participants with CD4 cell count between 51 and 200, and two participants with CD4>200 cells/mm^3^.

Of the five participants that were smear-positive but MGIT culture-negative for *M. tuberculosis*, three grew NTM. Table 2 shows Xpert results compared with LJ and MGIT culture.

**Table 2:** Comparison of Xpert MTB/RIF method with sputum culture.

Xpert MTB/RIF results by MGIT status of corresponding sputum specimen			
	MTB positive	NTM positive but MTB negative	Negative for NTM and MTB
Xpert MTB/RIF positive	94	3	7
Xpert MTB/RIF negative	29	16	275
**Xpert MTB/RIF results by LJ status of corresponding sputum specimen**			
Xpert MTB/RIF positive	89	1	14
Xpert MTB/RIF negative	15	2	303

**Key:** LJ  =  Lowenstein Jensen, MGIT  =  Mycobacterial Growth Indicator Tube, Xpert  =  Xpert MTB/RIF test, NTM  =  Non Tuberculous Mycobacteria.

### Diagnostic yield and sensitivity of the methods used for diagnosis of HIV-related tuberculosis

Among MGIT culture positive TB patients, sputum culture by LJ had the same yield as Xpert 104 (24.5%), however, LJ culture had a slightly higher sensitivity of 100 (81.3%) compared to Xpert test, 94 (76.4%) (Table 3). Xpert test detected slightly more TB cases than LJ, only among participants with CD4 cell count >200 cells/mm^3^ (Table 4).The sensitivity of Xpert decreased near-significantly from 91.7% (95% CI 73.0–98.9%) among participants with CD4 cell count >200 cells/mm^3^ to 73.2% (95% CI 63.2–81.7%) among participants with CD4 cell count ≤200 cells/mm^3^ (p = 0.062). There was almost no difference in sensitivity of Xpert between participants with CD4 cell count 50–200 cell/mm^3^ and participants with cell count <50 CD4 cell/mm^3^ (68.9% vs 76.9%, p = 0.491) (Table 5).

**Table 3:** Overall yield and sensitivity using MGIT as the reference comparator, for each of the test methods.

	overall (n = 424)		MGIT culture positive (n = 123)	
Test	Yield n (%)	95% CI	Sensitivity n (%)	95% CI
**DZN**	42 (9.9)	7.2–13.1	39 (31.7)	23.6–40.7
**DFM**	46 (10.8)	8.0–14.2	43 (35.0)	26.5–44.0
**CFM**	58 (13.7)	10.5–17.3	54 (43.9)	34.9–53.1
**Xpert**	104 (24.5)	20.5–28.9	94 (76.4)	67.9–83.6
**LJ**	104 (24.5)	20.5–28.9	100 (81.3)	73.2–87.7
**MGIT**	123 (29.0)	24.7–33.5	N/A	N/A

**Key:** Direct Ziehl Neelsen, DFM  =  Direct Fluorescent Microscopy, CFM  =  Concentrated Fluorescent Microscopy, LJ  =  Lowenstein Jensen, MGIT  =  Mycobacterial Growth Indicator Tube, Xpert  =  Xpert MTB/RIF test, CI  =  Confidence Interval.

**Table 4:** Overall yield of tests, by CD4 cell count at study enrollment (n =  419).

Test	>200 n (%)N = 138	95% CI	51–200 n (%)N = 117	95% CI	</ = 50 n (%)N = 164	95% CI
**DZN**	11 (8.0)	4.0–13.8	13 (11.1)	6.0–18.2	18 (11.0)	6.6–16.7
**DFM**	11 (8.0)	4.0–13.8	14 (12.0)	6.6–19.2	21 (12.8)	8.1–18.9
**CFM**	15 (10.9)	6.2–17.2	18 (15.4)	9.3–23.2	25 (15.2)	10.1–21.6
**Xpert**	25 (18.1)	12.1–25.5	34 (29.1)	21.0–38.1	44 (26.8)	20.2–34.2
**LJ**	19 (13.8)	8.4–20.6	37 (31.6)	23.3–40.8	46 (28.0)	21.3–35.5
**MGIT**	24 (17.4)	11.4–24.7	45 (38.5)	29.6–47.9	52 (31.7)	24.6–39.4

**Key:** Direct Ziehl Neelsen, DFM  =  Direct Fluorescent Microscopy, CFM  =  Concentrated Fluorescent Microscopy, LJ  =  Lowenstein Jensen, MGIT  =  Mycobacterial Growth Indicator Tube, Xpert  =  Xpert MTB/RIF test, CI  =  Confidence Interval.

**Table 5:** Sensitivity per test by CD4 cell count among MGIT confirmed TB cases (n = 121).

Test	>200 n (%)N = 24	95% CI	51–200 n (%)N = 45	95% CI	</ = 50 n (%)N = 52	95% CI
**DZN**	11 (45.8)	25.5–67.1	11 (24.4)	12.8–39.5	17 (32.7)	20.3–47.1
**DFM**	11 (45.8)	25.5–67.1	12 (26.7)	14.6–41.9	20 (38.5)	25.3–52.9
**CFM**	14 (58.3)	36.6–77.8	16 (35.6)	21.8–51.2	24 (46.2)	32.2–60.5
**Xpert**	22 (91.7)	73.0–98.9	31 (68.9)	53.3–81.8	40 (76.9)	63.1–87.4
**LJ**	19 (79.2)	57.8–92.8	36 (80.0)	65.4–90.4	43 (82.7)	69.6–91.7

**Key:** Direct Ziehl Neelsen, DFM  =  Direct Fluorescent Microscopy, CFM  =  Concentrated Fluorescent Microscopy, LJ  =  Lowenstein Jensen, MGIT  =  Mycobacterial Growth Indicator Tube, Xpert  =  Xpert MTB/RIF test, CI  =  Confidence Interval.

### Performance of Xpert test in an add-on strategy for TB diagnosis among HIV-infected participants

**Figure 2:**
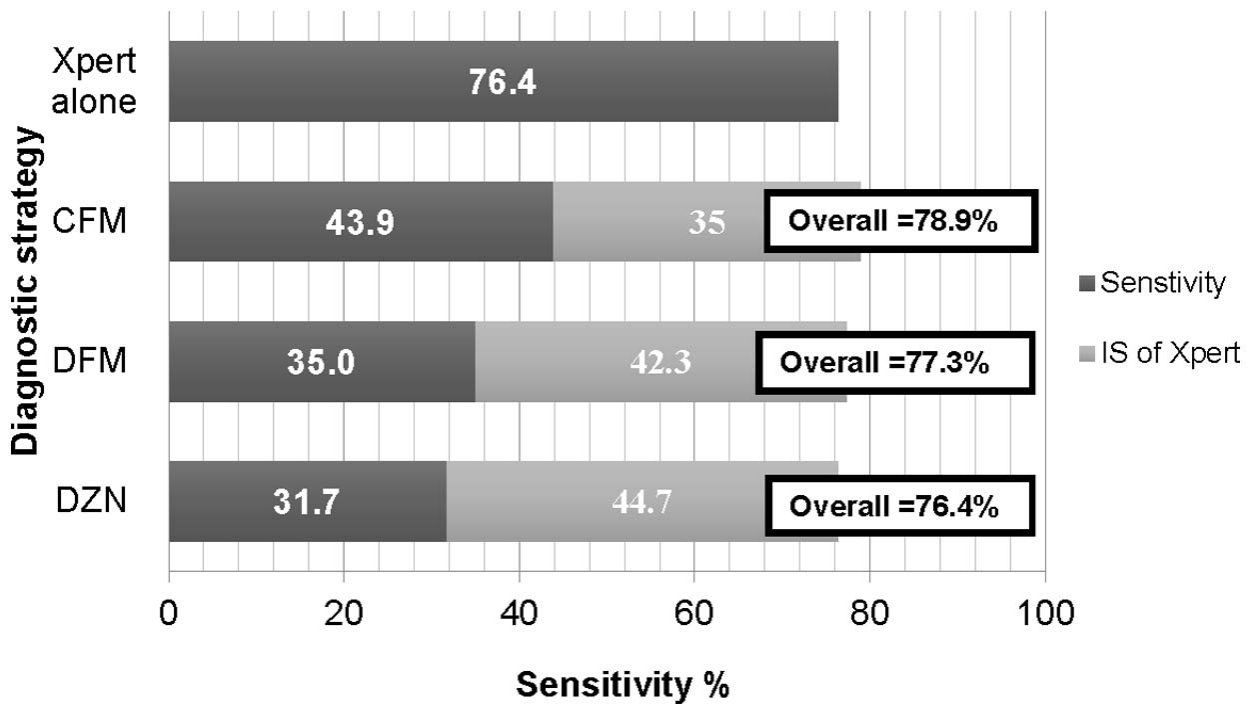
Sensitivity and incremental sensitivity of Xpert among smear negative MGIT culture confirmed TB cases (n = 123). DZN  =  Direct Zielh Neelsen, DFM  =  Direct Fluorescent Microscopy, CFM  =  Concentrated Fluorescent Microscopy, Xpert  =  Xpert MTB/RIF test, IS  =  Incremental sensitivity.

Xpert resulted in an incremental sensitivity (IS) of 55/123 (44.7%; 95% CI 35.7–53.9%) when added to DZN, 52/123(42.3%; 33.4–51.5%) when added to DFM and 43/123 (35.0%; 26.5–44.0%) when added to CFM. This translates into a combined sensitivity of 76.4%, 77.3% and 78.9% for Xpert done after a positive DZN, DFM and CFM smear examination, respectively, compared to the sensitivity of 76.4% for Xpert done independently (Figure 2).

In this study population one would, for the add-on strategies, need to do 424 smear examinations, plus 385 Xperts for DZN, 381 Xperts for DFM, and 370 Xperts for CFM, whereas for Xpert done independently one would do 424 Xpert tests. Using an add-on strategy one would thus require 9.2% (95% CI 6.6–12.3%) fewer Xpert tests when combined with DZN, 10.1% (7.4–13.4%) fewer Xpert tests when combined with DFM and 12.7% (9.7–16.2%) fewer Xpert tests when combined with CFM.

## Discussion

We have documented that among HIV-infected PTB suspects, an add-on strategy in which smear examination is done first and if negative followed by Xpert only identifies a few additional TB cases compared to a strategy in which Xpert is used as the first-line test. There is only 0–2.6% additional sensitivity for an add-on strategy and the proportion of Xpert tests saved by an add-on strategy is only around 10% (Figure 2). The smear examination method having the most sensitivity in an add-on strategy was FM after concentration by centrifugation, an elaborate method that generally cannot be performed in peripheral laboratories (Figure 2).

We found an increase in diagnostic gain from direct ZN to fluorescent microscopy that agrees with previous studies [6], [7], [27] (Table 3). Xpert detected more TB cases than any of the smear microscopy methods irrespective of concentration; moreover, smear microscopy is less specific as we have documented smear-positive participants with NTM in culture that were negative by Xpert. In agreement with previous studies [15], [16], this may be because for a positive result, Xpert requires fewer bacilli per mL of sputum sample leading to detection of TB cases that are likely to be missed by the smear examination methods that require more bacilli per mL, and Xpert targets gene sequences that are specific to *M. tuberculosis* [15], [28]. However, Xpert did give apparent false-positive results, including in 3 of 19 specimens that grew NTM but no *M. tuberculosis* on MGIT (Table 2). Although this seems to suggest that the specificity of Xpert for *M. tuberculosis* is below 100%, it may also be that the sensitivity of MGIT to detect M. tuberculosis in these patients was incomplete, or that the NTM-positive participants also had *M. tuberculosis*, which might have been outcompeted in MGIT culture [29], [30].

Sputum culture by LJ detected fewer cases than MGIT, as previously documented [31], [32]. LJ had the same yield as Xpert although it had slightly higher sensitivity (Table 3). The high diagnostic yield of Xpert obviates the need to do LJ culture, given the costly infrastructure and specialized staff requirements for the latter. Despite MGIT culture having a higher yield than the other methods used, it is more costly with more requirements for its use in routine TB diagnosis. Xpert assay also rapidly detected four rifampicin resistant participants. Using Xpert assay could thereby increase the number of individuals who are started timely on appropriate second-line treatment. Culture using MGIT detected more TB cases when compared with Xpert partly due to lower detection threshold in terms of number of bacilli per mL that is required for the MGIT culture to be positive as compared to a molecular assay like Xpert [33]. However, the long time to detection by culture that delays treatment initiation may not make it a better test for tuberculosis in individuals with HIV infection, who have high mortality if left untreated [34].

On the other hand and as previously documented [15], the sensitivity of Xpert, as well as of all smear examination methods per test decreased when CD4 cell counts decreased below 200 cells/mm^3^, while there was no further decrease in sensitivity when the CD4 cell count group of 50–200 cell/mm^3^ was compared to that of >50 cell/mm^3^ (Table 4 and Table 5). This trend emphasizes the need to use tests that are more sensitive in HIV-infected individuals with CD4 cell counts below 200 cell/mm^3^, such as molecular-based test and culture.

When we analyzed our findings in terms of incremental sensitivity after smear microscopy, the added benefit of combining Xpert with smear examination in an add-on strategy was small (Figure 2). In addition, there were only limited savings in terms of fewer Xpert tests done, whereas there will be additional costs and potential diagnostic delays due to the smear examinations.

The limited benefit of an add-on strategy could be related to the specific nature of this study population of HIV-infected individuals with on average low CD4 counts (two-thirds ≤200 cells/mm^3^). This reflects the likely operational cohort of HIV-infected smear-negative TB cases in Uganda and other low income countries where an add-on strategy is considered [21]. The sensitivity of smear examination of a single sputum sample from an HIV-infected individual is rather low (32–44%), whereas that of Xpert is relatively high. Moreover, one would not want to delay diagnosis of smear-negative TB in an HIV-infected individual because of the high mortality [34].

Our study had limitations. We performed Xpert on previously frozen processed sputum samples. However, previous studies showed that this is unlikely to produce different results from testing unprocessed samples [15]. Furthermore, we considered only one sputum samples in our analysis, which will have limited the sensitivity compared to testing two or more samples, including an early morning sample or in a frontloading system as generally recommended [31], [35], [36]. In practice however, often, a single sputum sample is available and we intended to evaluate the diagnostic value of add-on versus replacement strategies for that situation. We calculated sensitivity relative to MGIT culture, which is not 100% sensitive itself. Xpert positive and MGIT culture with NTM are likely to be true positives, underestimating the incremental sensitivity of Xpert. Moreover, NTM positive cultures were considered negative, among which Xpert identified additional TB cases. Finally, we used a rapid identification assay to confirm culture positive samples for MTB complex, however, previous studies have documented cases of false-negative results, which could have affected the discriminatory power of culture methods for NTM [37], [38]. Future studies with more robust identification methods such as 16s sequence based identification on smear positive antigen test negative cultures are recommended.

Our findings support a replacement strategy (i.e. Xpert only) for implementing Xpert for diagnosing TB among HIV-infected individuals if Xpert is available on-site. If Xpert is not available on-site, an add-on strategy may be helpful since, as in our study, 32–44% of HIV-infected individuals with TB may already be detected upon smear examination of a first sputum sample. These individuals, or their sputum specimens, then do not need to be referred to a clinic where Xpert tests can be performed, with obvious savings in terms of transport cost, delays and potentially dropout of the diagnostic process and related mortality. Indeed, despite WHO recommendations and subsidization of cartridges the availability at point-of-care level of Xpert will likely increase only slowly, given the cost of the equipment and importation of cartridges, and the difficulties in resource-constrained settings with machine calibration and maintenance. An add-on strategy in which smear negative samples are referred for Xpert testing will only be economically efficient (transport, sample packaging, time-wise) if Xpert machines are decentralized to testing hubs within the district or to district reference laboratories. Further studies are needed to evaluate the cost-effectiveness of various strategies for implementing Xpert under routine, programmatic conditions before being scaled-up in resource-limited settings.

## Supporting Information

Table S1 **Comparative performance of Xpert, LJ and MGIT by smear microscopy status for diagnosis of HIV-related tuberculosis.**
(DOC)
