# Supplementary material for: Clinical Utility of a Novel Molecular Assay in Various Combination Strategies with Existing Methods for Diagnosis of HIV-Related Tuberculosis in Uganda
Source: PLoS One. 2014 Sep 15;9(9):e107595. doi: 10.1371/journal.pone.0107595 (PMC4164637; doi:10.1371/journal.pone.0107595)
Supplement: Table S1 — Comparative performance of Xpert, LJ and MGIT by smear microscopy status for diagnosis of HIV-related tuberculosis. (DOC) [file pone.0107595.s001.doc]

**Table S1. Comparative performance of Xpert, LJ and MGIT by smear microscopy status for diagnosis of HIV-related tuberculosis**

| **Sub sets ( Smear Positive)** | **DZN** | **DFM** | **CFM** |
| --- | --- | --- | --- |
| Xpert +, LJ +, MGIT+ | 36 | 39 | 47 |
| Xpert +, LJ +, MGIT - | 0 | 0 | 0 |
| Xpert +, LJ -, MGIT + | 3 | 3 | 5 |
| Xpert +, LJ -, MGIT - | 1 | 1 | 1 |
| Xpert -, LJ +, MGIT + | 1 | 2 | 3 |
| Xpert -, LJ +, MGIT - | 0 | 0 | 0 |
| Xpert -, LJ-, MGIT + | 0 | 0 | **1*** |
| Xpert -, LJ -, MGIT - | 1 | 1 | 1 |
| **Sub sets (Smear Negative)** | - | - | - |
| Xpert +, LJ +, MGIT+ | 54 | 51 | 43 |
| Xpert +, LJ +, MGIT - | 0 | 0 | 0 |
| Xpert +, LJ -, MGIT + | 4 | 4 | 2 |
| Xpert +, LJ -, MGIT - | 6 | 6 | 6 |
| Xpert -, LJ +, MGIT + | 16 | 15 | 14 |
| Xpert -, LJ +, MGIT - | 0 | 0 | 0 |
| Xpert -, LJ-, MGIT + | 28 | 28 | 27 |
| Xpert -, LJ -, MGIT - | 274 | 274 | 274 |
| **Total patterns** | **424** | **424** | **424** |

**Key:** *= All positive, underlined, plus 1(*), n =123 positive participants, DZN = Direct Zielh Neelsen, DFM = Direct Fluorescent Microscopy, CFM = Concentrated Fluorescent Microscopy, Xpert = Xpert MTB/RIF test
